# Loss of *Jagged1* in renin progenitors leads to focal kidney fibrosis

**DOI:** 10.14814/phy2.12544

**Published:** 2015-11-04

**Authors:** Brian C Belyea, Fang Xu, Maria Luisa S Sequeira-Lopez, R Ariel Gomez

**Affiliations:** Department of Pediatrics, University of Virginia School of MedicineCharlottesville, Virginia

**Keywords:** jagged1, notch, renin

## Abstract

The Notch signaling pathway is required to maintain renin expression within juxtaglomerular (JG) cells. However, the specific ligand which activates Notch signaling in renin-expressing cells remains undefined. In this study, we found that among all Notch ligands, *Jagged1* is differentially expressed in renin cells with higher expression during neonatal life. We therefore hypothesized that *Jagged1* was involved in renin expression and/or vascular integrity. We used a conditional knockout approach to delete *Jagged1* in cells of the renin lineage. Deletion of *Jagged1* specifically within renin cells did not result in decreased renin production within the kidney. However, animals with conditional deletion of *Jagged1* did develop focal kidney fibrosis and elevated blood urea nitrogen. Our data demonstrate that Jagged1-mediated Notch signaling is dispensable in renin cells of the kidney in regard to renin expression. However, deletion of *Jagged1* in renin cells descendants affects perivascular–interstitial integrity leading to focal fibrosis and diminished renal function.

## Introduction

Renin-expressing cells are crucial in the control of blood pressure and fluid-electrolyte homeostasis and nephrovascular development (Gomez et al. 2014). While the mechanisms that maintain renin expression have not been fully identified, they are likely dependent on the direct interaction between renin and adjacent cells. The Notch signaling pathway is an evolutionarily conserved pathway, which is vital for cell–cell communication during fundamental developmental processes (Tanigaki and Honjo 2010). Members of the Notch signaling pathway, including receptors, ligands, and the final common effector, *RBP-J*, are expressed in renin cells of the kidney (Brunskill et al. 2011). Therefore, we hypothesized that the Notch signaling pathway, conveyed by cell–cell signaling, is important in maintaining the renin cell identity. To that end, our laboratory previously used a conditional knockout approach to delete the final effector of Notch signaling, *RBP-J*, specifically within cells of the renin lineage (Castellanos Rivera et al. 2011). Conditional deletion of *RBP-J* in renin cells resulted in a severe reduction in the number of renin-positive cells in the kidney. Furthermore, it was determined that this effect is via canonical Notch signaling, as treatment of mice with a gamma secretase inhibitor (which represses the Notch pathway) nearly phenocopies the effect of *RBP-J* deletion (unpublished work from our laboratory). Together, this work showed that Notch/RBPJ signaling is required to maintain renin expression within juxtaglomerular (JG) cells. However, how the Notch pathway is activated in renin cells remains undefined. Therefore, we questioned which Notch ligand is responsible for providing the instructive cues to renin cells, triggering the Notch signaling pathway, and thus responsible for maintaining the identity of renin cells and their descendants. In this study, we investigated the expression of the Notch ligand *Jagged1* in renin cells and the effects of conditional *Jagged1* deletion.

## Materials and Methods

### Mice

To investigate whether expression of *Jagged1* is critical for renin cell development, we generated mice with conditional deletion of *Jagged1* specifically in renin cells. We crossed *Jagged1*^*fl/fl*^ mice (Kiernan et al. 2006) which contain LoxP sites flanking exon 4, the DSL domain of the *Jagged1* gene, with EIIa-Cre mice which express *cre* in the early embryo, resulting in germline deletion of the loxP-flanked *Jagged1* allele (*Jagged1*^*del/+*^) (Lakso et al. 1996). These mice were then crossed with *Jagged1*^*fl/fl*^ mice and *Ren1*^*d cre/+*^ mice (Sequeira-Lopez et al. 2004), ultimately generating mice with one deleted *Jagged1* allele, one floxed *Jagged1* allele, and one copy of *Ren1*^*d cre/+*^ (*Jagged1*^*del/fl*^*;Ren1*^*d cre/+*^) termed “mutant” animals in this study. Control animals either lacked *cre recombinase* (*Jagged1*^*del/fl*^*;Ren1*^*d +/+*^) or had a wild-type allele of *Jagged1* (*Jagged1*^*+/fl*^*;Ren1*^*d cre/+*^) and are termed “control” animals in this study. *Ren1*^*d cre*^ animals were generated previously in our laboratory (Sequeira-Lopez et al. 2004), and *Jagged1*^*fl/fl*^ mice were obtained from Jackson Laboratories. All procedures were performed following the National Institutes of Health *Guide for the Care and Use of Laboratory Animals* and were approved by the University of Virginia Animal Care and Use Committee.

### Genotyping

Tail biopsies were sent to Transnetyx for genotyping (Transnetyx Genotyping Services, Cordova, TN).

### Histological and immunohistochemical analysis

Mice were anesthetized with tribromoethanol (300 mg/kg). Kidneys were harvested at indicated time points, weighed, fixed overnight in Bouin’s fixative, and embedded in paraffin. Sections (5 *μ*m) were deparaffinized in xylenes and graded alcohols and stained with hematoxylin and eosin to examine overall kidney morphology and Masson’s Trichrome to identify areas of fibrosis as described previously (Gomez et al. 2009; Sequeira-Lopez et al. 2010).

We performed immunohistochemistry for renin (1:500 dilution of rabbit polyclonal anti-mouse antibody) to determine the distribution of renin-expressing cells, *Jagged1* (1:100 dilution of goat polyclonal antibody; Santa Cruz, Dallas, TX) to demonstrate conditional deletion, and *α*-smooth muscle actin (SMA, 1:10,000 dilution of mouse monoclonal antibody; Sigma, St. Louis, MO) to evaluate vascular architecture, as described previously (Sequeira-Lopez et al. 2001; Lin et al. 2014). To quantitate the occurrence of fibrosis in control and mutant animals, we reviewed midsagittal sections of kidneys from control and mutant animals at each of the indicated ages. Fibrosis was scored as areas of increased *α*SMA staining of interstitial cells and dilated tubules.

The juxtaglomerular apparatus (JGA) index was calculated as the number of renin-positive JGA per total number of glomeruli and expressed as a percentage. To determine the length of renin staining, we measured the maximal length of each renin-staining JGA.

### RNA extraction and PCR analysis

Whole kidneys from control and mutant animals were removed and placed in RNA*later* (Ambion, Austin, TX) overnight in 4°C and then stored in −20°C. Total RNA was isolated using Trizol extraction (Life Technologies, Grand Island, NY) according to the manufacturer’s instructions. cDNA was prepared from 2 *μ*g of RNA using Maloney murine leukemia virus reverse transcriptase (Life Technologies, Grand Island, NY) and an oligo(dT) primer according to the manufacturer’s instructions. Quantitative reverse transcription polymerase chain reaction (RT-PCR) was performed using SYBR Green I (Invitrogen Molecular Probes, Eugene, OR) in a CFXConnect system (BioRad, Hercules, CA). Renin and *Jagged1* mRNA expression was normalized to GAPDH expression, and the changes in expression were determined by the ∆∆Ct method and are reported as relative expression compared to control mice. The following primers were used: renin forward 5’-ACAGTATCCCAACAGGAGAGACAAG-3’ and renin reverse 5’-GCACCCAGGACCCAGACA-3’; *Jagged1* forward 5’-GCAACGACCGTAATCGCATC-3’ and *Jagged1* reverse 5’-CCATTGCCGGCTAGGGTTTA-3’; GAPDH forward 5’-TTGATGGCAACAATCTCCAC-3’ and GAPDH reverse 5’-CGTCCCGTAGACAAAATGGT-3’.

### Plasma renin

Plasma was collected from animals at the time of sacrifice. Plasma renin concentration was determined using Renin ELISA following the manufacturer’s instructions (RayBiotech, Norcross, GA).

### Statistical analysis

Values are expressed as mean ± standard error of the mean (SEM). Statistical significance between groups was evaluated using Student’s *t*-test and two-way analysis of variance. Differences were considered statistically significant at *P < 0.05, **P < 0.01, and ***P < 0.001 levels.

## Results

### *Jagged1* is expressed in renin cells during kidney development

**Figure 1:**
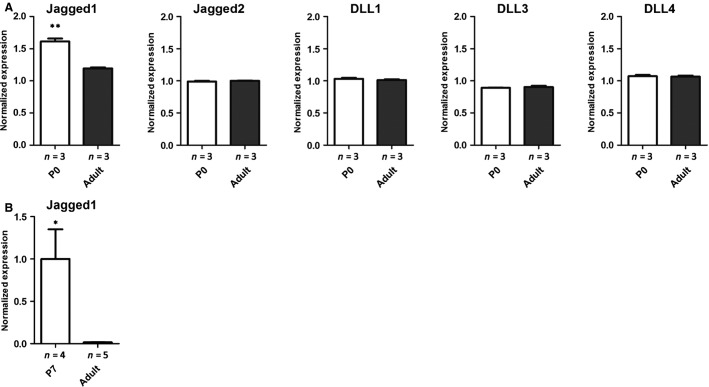
*Jagged1* is upregulated in renin cells during neonatal life compared to adult. (A) The expression of all five Notch ligands within renin cells at P0 and adult ages was assessed from a publicly available database (Brunskill et al. 2011) (**P < 0.01, *n* = 3 for each condition). (B) Quantitative PCR for *Jagged1* was performed at P7 and adult ages (*P < 0.05, *n* = 4 for P7 and *n* = 5 for adult time points).

To determine which Notch ligands are expressed in renin cells during kidney development, we first examined previously published and publicly available gene microarray data generated in our laboratory for expression of the Notch ligands *Jagged1*, *Jagged2*, *DLL1*, *DLL3*, and *DLL4* within JG cells (Brunskill et al. 2011). This analysis demonstrated that *Jagged1* is upregulated in the renin cells during neonatal life compared to adult (Fig.1A). Furthermore, *Jagged1* is the most highly expressed gene of the five Notch signaling ligands within renin cells.

We then performed quantitative RT-PCR on RNA extracted from whole kidney to confirm the expression pattern of *Jagged1* in neonatal and adult kidney. This demonstrated increased expression of *Jagged1* shortly after birth with decreasing expression with age (Fig.1B). Together, these data demonstrate that *Jagged1* is expressed in renin cells of the kidney during neonatal life and suggests this ligand may be the key pathway member triggering Notch signaling.

### Deletion of *Jagged1* in renin cells

**Figure 2:**
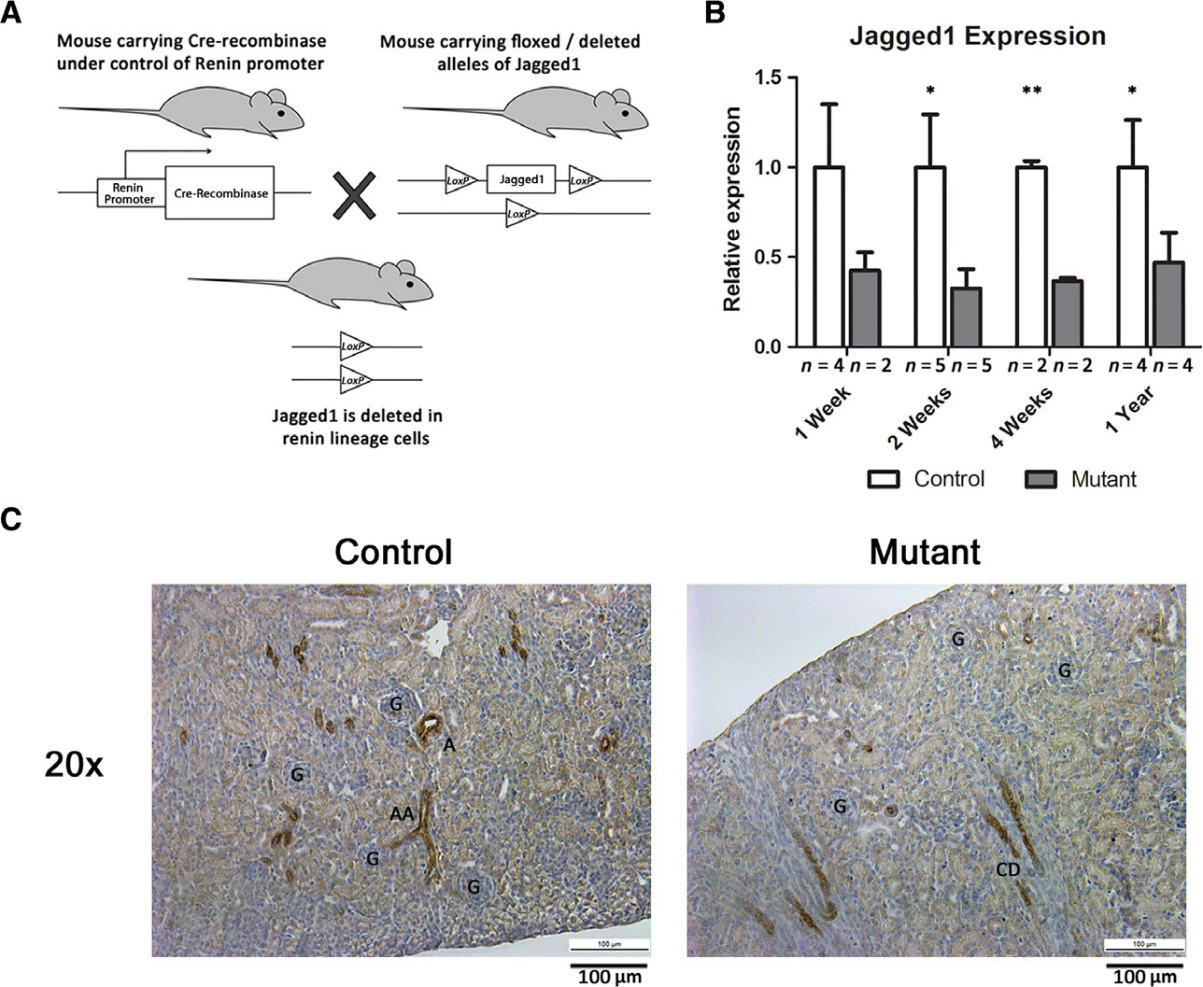
Deletion of *Jagged1* within renin cells. (A) A schematic of the breeding strategy for conditional deletion of *Jagged1* in renin cells. (B) *Jagged1* deletion efficiency as measured by quantitative PCR. There was >50% reduction in *Jagged1* expression levels at each time point (*P < 0.05; **P < 0.01, *n* = 4, 5, 2, and 4 for control and 2, 5, 2, and 4, for mutant kidneys at 1 week, 2 weeks, 4 weeks, and 1 year, respectively). (C) Immunostaining for *Jagged1* (brown staining) in 1-week-old control and mutant kidney sections. Scale bar: 100 *μ*m. G = glomeruli, A = artery, AA = afferent arteriole, CD = collecting duct.

To investigate whether expression of *Jagged1* is critical for renin cell development, we generated mice with conditional deletion of *Jagged1* specifically in renin cells. We crossed mice that express *Cre recombinase* under control the renin locus (Sequeira-Lopez et al. 2004) with mice that have the *Jagged1* allele either deleted or floxed (see Materials and Methods section) (Lakso et al. 1996; Kiernan et al. 2006) (Fig.2A). Control mice (*Jagged1*^*+/fl*^*;Ren1*^*d cre/+*^ or *Jagged1*^*del/fl*^*;Ren1*^*d +/+*^) and mutant mice (*Jagged1*^*del/fl*^*;Ren1*^*d cre/+*^) were then studied at indicated time points.

To assess the effectiveness of *Jagged1* deletion, we performed quantitative RT-PCR for *Jagged1*. Compared to control mice, expression of *Jagged1* mRNA was reduced in mutant mice at 7 days, 2 weeks, 4 weeks, and 1 year of age as shown by quantitative PCR studies (Fig.2B). Of note, RNA was extracted from whole kidney, and thus the efficiency of *Jagged1* deletion specifically within renin cells may be underestimated.

In addition, we performed immunohistochemistry for *Jagged1* in kidney sections of control and mutant animals. We examined *Jagged1* immunostaining at P7, because *Jagged1* is at its peak expression during neonatal life and it is easier to observe differences between control and mutant animals, whereas later in life, *Jagged1* expression is downregulated. At 1 week of life, we found that there was *Jagged1* staining in the arteries, afferent arterioles, and JG cells as well as collecting ducts of control mice. However, in mutant mice, there was decreased *Jagged1* staining in the afferent arterioles and JG regions (Fig.2C). In both control and mutant kidneys, *Jagged1* is expressed in the collecting ducts, which may be the reason we see only 50–60% reduction in *Jagged1* mRNA levels in whole kidneys from mutant mice. Together, these data demonstrate conditional deletion of *Jagged1* in renin-expressing and renin-lineage cells.

### Renin expression is unaffected by deletion of *Jagged1* in renin cells

**Figure 3:**
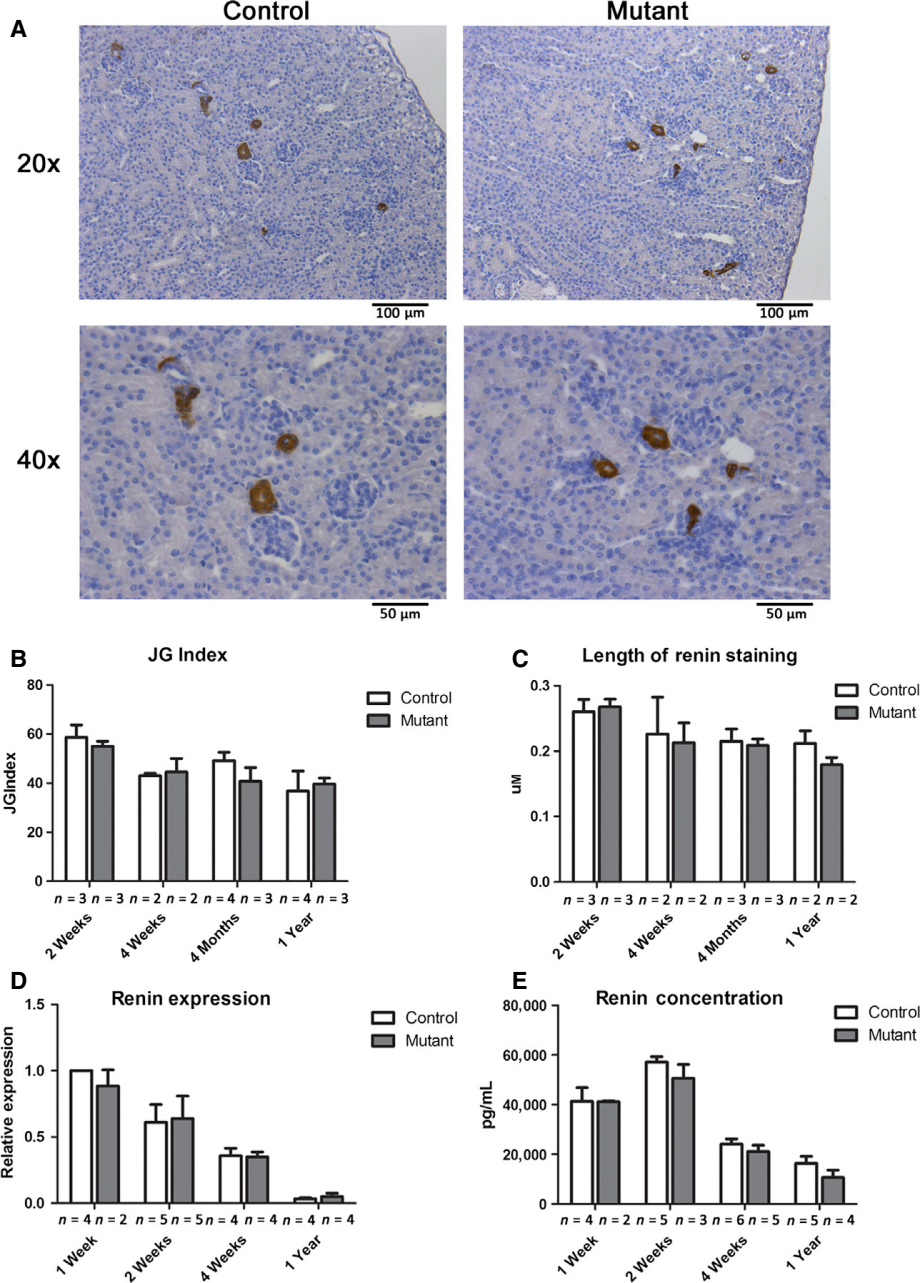
Renin expression is unaffected by deletion of *Jagged1* in renin cells. (A) Immunostaining for renin (brown staining) in 2-week-old control and mutant kidney sections at low and high magnification. Scale bar: 100 *μ*m top panel and 50 *μ*m bottom panel. (B) Quantification of the number of juxtaglomerular (JG) apparatuses that stain positive for renin demonstrates no difference between control and mutant kidneys at indicated time points (*n* = 3, 2, 4, and 4 for control animals and *n* = 3, 2, 3, and 3 for mutant animals at 2 weeks, 4 weeks, 4 months, and 1 year, respectively). (C) The length of renin staining was measured from positively stained JG cells along the afferent arteriole. A minimum of 12 measurements were taken for each sample (*n* = 3, 2, 3, and 2 for both control and mutant kidneys at 1 week, 2 weeks, 4 weeks, and 1 year, respectively). (D) Relative renin mRNA levels for control and mutants animals (*n* = 4, 5, 4, and 4 for control animals and *n* = 2, 5, 4, and 4 for mutant animals at 1 week, 2 weeks, 4 weeks, and 1 year, respectively). (E) Circulating plasma renin levels in control and mutant animals (*n *= 4, 5, 6, and 5 for control animals and *n* = 2, 3, 5, and 4 for mutant animals at 1 week, 2 weeks, 4 weeks, and 1 year, respectively).

Control and mutant kidneys were sectioned and stained for renin, and the number of renin-positive JGA was examined (Fig.3A). There was no reduction in the number of renin-positive JGAs when corrected for the total number of glomeruli (JGA index) in mutant animals compared to controls at 2 weeks, 4 weeks, 4 months, and 12 months (Fig.3B). Renin production can be increased through the recruitment of additional renin-synthesizing JG cells (which would increase the JG index) or through recruitment of smooth muscle cells along the afferent arteriole (which would not be reflected by the JG index). Therefore, we measured the length of renin staining from control and mutant animals to determine if there was decreased extension of staining in mutants. There was no significant difference between control and mutant animals (Fig.3C).

In agreement with these data, there was also no difference in renin mRNA expression in whole kidney as measured by qRT-PCR between control and mutant animals at 7 days, 2 weeks, 4 weeks, and 1 year of age (Fig.3D). Finally, the concentration of circulating renin was measured in the plasma of control and mutant mice, and there was no difference in the levels of renin at the same time points (Fig.3E). As expected, renin expression and circulating levels of renin decreased with age in both control and mutant animals (Gomez et al. 1991). Together, these data demonstrate that conditional deletion of *Jagged1* within renin cells does not result in diminished renin production.

### Deletion of *Jagged1* in renin cells leads to focal fibrosis

**Figure 4:**
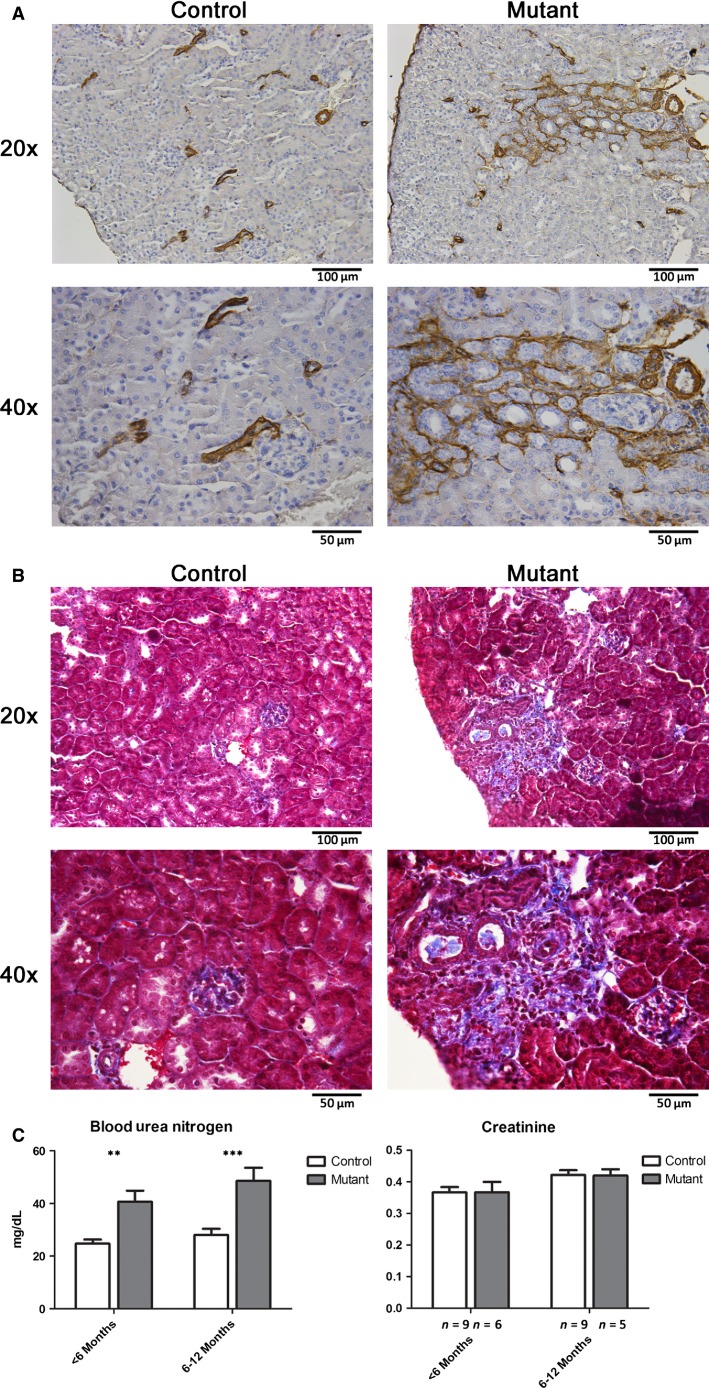
Deletion of *Jagged1* in renin cells leads to focal areas of fibrosis and decreased renal function. (A) Immunostaining for *α*-smooth muscle actin (*α*SMA) demonstrates focal areas of injured interstitial cells and dilated tubules which express *α*SMA in mutant animals (age 4 months). (B) Masson’s Trichrome staining demonstrates areas of increased collagen fibers in mutant animals (age 4 months). (C) Blood urea nitrogen and creatinine measured from peripheral blood of control and mutant animals at indicated ages (**P < 0.01; ***P < 0.001, *n* = 9 and 9 for control animals and *n* = 6 and 5 for mutant animals at age <6 months and 6–12 months, respectively).

Immunostaining for *α*SMA demonstrated an overall normal vascular anatomy in both control and mutant kidneys. However, in mutant kidneys, there were focal areas containing activated interstitial cells and dilated tubules expressing *α*SMA, indicating an active fibrotic process (Fig.4A). Masson’s trichrome staining demonstrated increased collagen fibers in mutant kidneys, consistent with areas of interstitial fibrosis (Fig.4B). Overall, we found an increased incidence of focal areas of fibrosis in mutant animals compared to control animals, and this appeared to worsen with age (Table1). In accordance with the presence of focal fibrosis, we also found that mutant animals had decreased renal function as measured by blood urea nitrogen, however with preserved creatinine (Fig.4C). Similar to the pattern of focal fibrosis, renal function appeared to worsen with age.

**Table 1:** Frequency of focal kidney fibrosis in control and mutant animals

Age	Control	Mutant
2 Weeks	0/3	1/3
4 Weeks	0/5	2/5
4 Months	0/4	4/4
1 Year	1/5	2/3

## Discussion

In this study, we found that the Notch ligand *Jagged1* is expressed in renin cells during neonatal life. Conditional deletion of *Jagged1* specifically within renin cells did not result in decreased renin expression in JG cells. However, animals with loss of *Jagged1* in renin cells did develop focal kidney fibrosis and decreased renal function.

The Notch pathway is a highly conserved signaling pathway which plays a critical role in cell fate decisions in a variety of tissue and cell types (Andersson et al. 2011). In mammals, there are five Notch ligands (Jagged1, 2, DLL1, 3, 4) and four Notch receptors (Notch1–4). The Notch pathway is activated when two cells come into contact, facilitating Notch ligand–receptor binding between adjacent cells and leading to liberation of the intracellular component of Notch (ICN). ICN then translocates to the nucleus where it forms a transcriptional activation complex with RBP-J and ultimately influences Notch target gene expression. Previous studies from our laboratory and others have demonstrated that the Notch signaling pathway plays a crucial role in renin cell development and regulation (Pan et al. 2005; Castellanos Rivera et al. 2011, 2015). Conditional deletion of *RBP-J*, the central mediator of Notch signaling, results in decreased renin expression (Castellanos Rivera et al. 2011). However, which Notch ligand and receptor provide the signal to *RBP-J* remains unknown. In this work, we found that the Notch ligand *Jagged1* is more highly expressed in renin cells during development when compared to other Notch ligands. Thus, we hypothesized that Jagged1-initiated juxtacrine signaling between neighboring renin cells plays a crucial role in renin cell development. Surprisingly, conditional deletion of *Jagged1* had no effect on renin expression, suggesting that Jagged1-mediated Notch signaling is dispensable for renin expression within JG cells. It is possible that other Notch ligands may compensate for loss of *Jagged1* within renin cells. Indeed, a precedent for redundancy in Notch signaling has been demonstrated in other tissue and organ systems. Demehri and Kopan (2009) demonstrated that deletion of a single notch receptor within hair follicle stem cells was insufficient to cause disease, however deletion of multiple receptors created a severe phenotype of follicle destruction which phenocopied deletion of the common effector *RBP-J*. Thus, it is possible that inhibition of the Notch pathway in renin cells is dosage dependent, where deletion of a single ligand (or receptor) is compensated, but deletion of multiple components or the final common effector *RBP-J* creates more striking disease. Further studies will be necessary to determine if other Notch ligands compensate for loss of *Jagged1* in preserving renin expression and if deletion of multiple Notch ligands (or receptors) in renin cells will have a more dramatic phenotype.

The pathogenesis of the focal fibrosis seen in this model is not completely understood, however, it is likely due to abnormal differentiation of renal vessels within the kidney leading to areas of decreased perfusion and hypoxia. In addition to the role of renin-synthesizing cells in control of blood pressure and fluid–electrolyte homeostasis, renin progenitor cells are precursors for a subset of arteriolar smooth muscle cells. Thus, deletion of *Jagged1* in renin precursors may lead to abnormal differentiation of renal vessels, then patchy hypoperfusion, ischemia, and ultimately fibrosis. More work will be needed to elucidate the mechanism of fibrosis, however these findings suggest an important role of *Jagged1* in the maintenance of the morphologic integrity of the kidney vasculature and parenchyma.

## Supporting Information

**Table S1.** The raw data (markers of kidney function in control and mutant animals) for Figure 4C.
